# Recent Developments in Alcohol Services Research on Access to Care

**DOI:** 10.35946/arcr.v38.1.04

**Published:** 2016

**Authors:** Laura A. Schmidt

**Affiliations:** Laura A. Schmidt, Ph.D., M.S.W., M.P.H., is a professor at the Philip R. Lee Institute for Health Policy Studies and the Department of Anthropology, History, and Social Medicine at the School of Medicine, University of California at San Francisco, San Francisco, California

**Keywords:** Alcohol use disorder, alcohol services research, health care disparities, health care financing, treatment, substance abuse treatment, treatment access, access to care, parity, socioeconomic disparity, special populations, gender, age, race, ethnicity, health care reform, Patient Protection and Affordable Care Act

## Abstract

In the United States, only about 10 percent of people with an alcohol or drug use disorder receive care for the condition, pointing to a large treatment gap. Several personal characteristics influence whether a person will receive treatment; additionally, many people with an alcohol use disorder do not perceive the need for treatment. The extent of the treatment gap differs somewhat across different population subgroups, such as those based on gender, age, or race and ethnicity. Recent health care reforms, such as implementation of the Patient Protection and Affordable Care Act of 2010, likely will improve access to substance abuse treatment. In addition, new treatment approaches, service delivery systems, and payment innovations may facilitate access to substance abuse services. Nevertheless, efforts to bridge the treatment gap will continue to be needed to ensure that all people who need alcohol and drug abuse treatment can actually receive it.

Of the more than 18 million Americans who need treatment for alcohol use disorder (AUD), less than 10 percent actually receive care (Substance Abuse and Mental Health Services Administration [SAMHSA] 2013). This problem, often referred to as the substance abuse treatment gap, is a longstanding concern for alcohol services research. Studies suggest that many factors contribute to the treatment gap, ranging from inadequate treatment capacity to organization and financing policies, negative attitudes on the part of potential treatment seekers, and inequities in the distribution of care. However, today, the landscape of alcohol treatment is shifting with health care reform, the advent of new treatment modalities, and secular changes in the populations needing care. In light of these trends, the research and treatment communities are seeking new answers to old questions: What is the current scope and nature of the treatment gap? Which subpopulations are the most underserved? How are major policy changes affecting access to alcohol treatment? And how can the newest treatments become available to a wider segment of the population in need?

## Understanding the Treatment Gap

Recent analyses of the U.S. population buttress claims that there exists a considerable unmet need for substance abuse treatment—enough to warrant serious, sustained attention by policymakers. It is safe to say that the substance abuse treatment gap in the United States is somewhere close to 90 percent. In other words, only about 10 percent of people with a current alcohol or drug use disorder receive care for the condition. This conclusion is based on a thorough national analysis that estimated the treatment gap using a wide range of possible metrics (Schmidt 2007*a*). The analysis found that even after using diverse measurement approaches, estimates of the treatment gap tended to cluster within a relatively narrow range of 8 percent to 12 percent. More recently, the 2014 National Survey on Drug Use and Health (NSDUH) found that approximately 18 percent of people needing treatment for alcohol and other drug use problems actually received any care in the previous year, and about 11 percent received specialty care (SAMHSA 2015). These estimates of the change in treatment gap pale in comparison to the magnitude of the problem they quantify.

The substantial gap between those who need treatment and those who actually get treatment has, in fact, been a longstanding issue in alcohol services research. In the 1980s, researchers began trying to understand what distinguished people who receive treatment from those who do not (Weisner 1988). What began as an effort to simply describe the problem evolved into a wide-ranging research enterprise seeking to explain why so many Americans fail to obtain needed care. Further analyses demonstrated that a cluster of factors robustly predict the likelihood of receiving substance abuse treatment, including the client’s age, gender, marital status, perceived need for treatment, and prior use of services (Weisner et al. 2002).

It also is clear that people who meet the criteria for an AUD often do not see a need for professional care. According to the 2014 NSDUH, only 6.3 percent of people diagnosed with substance use disorder or treated for substance use problems in a specialty treatment facility felt that they needed treatment (SAMHSA 2015), and the majority did not make an effort to seek care (SAMHSA 2015). Respondents cited several reasons for not seeking or receiving treatment, including not being ready to stop substance use, lack of health care coverage or means to afford treatment, fear of problems at work or stigmatization by others, and not knowing where to go for treatment. Others may question the efficacy of treatment (SAMHSA 2002). However, the reaction of family and friends to a person’s drinking problem can motivate care seeking, even when the affected individual is hesitant, and social support also can influence responses to treatment (Worley et al. 2015).

Some investigators have examined the “thresholds of severity” at which individuals with a drinking problem will perceive a need for care (Schmidt 2007*a*). These studies found that a person who is experiencing symptoms of mental distress, in addition to having problems with substance use, is much more likely to see a need for treatment than is a person without those symptoms. Once again, perceptions by others in the problem drinker’s life are critical factors in seeking care. Experiencing family, work, and legal problems also significantly increase the likelihood that people would see a need for care and eventually get there.

## Who Lacks Care? Uneven Access Across Subpopulations

Not all subgroups in the U.S. population are equally affected by the treatment gap. To better understand the causes and extent of the treatment gap for people with AUD, it is useful to look separately at different subpopulations based on gender, age, race and ethnicity, and other variables.

### Gender

During the 1980s, women were under-represented in addiction treatment programs by a one-to-four ratio compared with men. Therefore, researchers prodigiously investigated the reasons contributing to this underrepresentation, finding that women largely sought care from other types of providers, such as mental health providers, to avoid the stigma of substance abuse treatment (Weisner and Schmidt 1992). Since then, the gender gap has substantially narrowed (Steingrímsson et al. 2012). Although almost twice as many men than women received any substance use treatment in 2014 (Center for Behavioral Health Statistics and Quality 2015), the prevalence of substance abuse and dependence similarly was about twice as high among men as it was among women.^1^ The narrowing of this gender gap has led researchers to focus on other underserved populations.

### Age

A significant concern today is the disproportionately low rate of treatment utilization, and particularly specialty treatment, among adolescents and young adults in the United States. According to the 2014 NSDUH, about 1.3 million adolescents ages 12–17, and 5.8 million young adults ages 18–25, needed treatment for substance use problems (SAMHSA 2015). However, only 8.5 percent of these adolescents and 8.0 percent of young adults received treatment at a specialty facility, compared with 13.2 percent of adults ages 26 and older who needed treatment (SAMHSA 2015). The need for treatment appears similar among male and female adolescents, as indicated by a similar prevalence of substance abuse and dependence, but females are more likely to receive care from professionals specially trained in substance abuse treatment (Center for Behavioral Health Statistics and Quality 2015).

Looking at the other end of the age spectrum, studies point to a treatment gap for elderly people with alcohol and illicit drug problems, albeit a narrower one. According to the 2014 NSDUH, more than 1.1 million people ages 65 and older needed treatment for a substance use disorder, but only about 234,000 people in this age group (or about 21 percent) received treatment (Center for Behavioral Health Statistics and Quality 2015). This treatment gap may, at least in part, result from difficulties with the identification and diagnosis of substance use problems in this population (Blow et al. 2002).

### Race and Ethnicity

The debate about racial and ethnic disparities in health care access reached national prominence in 2002, with the publication of the watershed Institute of Medicine report *Unequal Treatment: Confronting Racial and Ethnic Disparities in Health Care* (Smedley et al. 2002). The report delivered a scathing view of gross inequities in access to, and the quality of, health care for America’s racial and ethnic minority groups. Although it seemed almost inevitable that substance abuse researchers would uncover similar evidence of disparities, by and large, those observed in the wider health care system appear far more pronounced.

Studies in the substance abuse field show more modest and subtle variations in treatment access by race and ethnicity (Schmidt et al. 2006). African Americans and Hispanics—the two groups most commonly studied—tend to experience more health and social consequences for a given level of drinking than their White counterparts. The higher incidence of negative social consequences among minorities could result from stress associated with discrimination or from differences in how various racial and ethnic communities respond to risky drinking and how the wider society responds to drinking within these communities (Mulia et al. 2009). With respect to treatment use, few differences exist between Whites, African Americans, and Hispanics, at least in those who experience alcohol problems on the less severe end of the spectrum. With increasing problem severity, however, African Americans and Hispanics have lower odds of entering treatment compared with Whites (Chartier and Caetano 2010; Schmidt et al. 2007*b*). In addition, when members of different ethnic groups do seek help for an alcohol problem, they tend to obtain different types of care. Hispanics receive less specialty care than do Whites (Schmidt et al. 2007*b*). Finally, although treatment retention is similar across ethnic groups, White patients receive more types of clinical services than Hispanics or African Americans, with the exception that African Americans receive more employment services (Niv et al. 2009).

One potential contributor to ethnic disparities in treatment access is geographic variation in the availability of treatment slots. In an interstate comparison of the alcohol treatment supply, McAuliffe and Dunn (2004) found that the Southern and South-western regions of the United States—regions with disproportionately large minority populations—are the most underserved. Surveys suggest that long wait times resulting from limited treatment capacities are a primary reason for unmet treatment need (Andrews et al. 2013). In national surveys, African Americans were disproportionately more likely to report lengthy wait times as a reason for not entering care (Schmidt et al. 2006). Individuals referred to treatment by the criminal justice system, who are more likely to belong to a minority group, also experience longer wait times (Andrews et al. 2013).

## Who Pays? Health Care Reform, Parity, and Access to Care

Lack of or insufficient insurance coverage may be one of the barriers that prevents people with alcohol problems from entering treatment. Accordingly, recent health care reforms are expected to have a significant impact on access to substance abuse treatment. In the late 1990s and early 2000s, mental health and substance abuse spending was growing at a slower rate than the gross domestic product and shrinking as a share of all health care spending (Mark et al. 2011). Indications are that this could change dramatically under health care reform. Approximately 25 million individuals will become newly insured as a result of the Patient Protection and Affordable Care Act of 2010 (ACA), known colloquially as “Obamacare” (Mark et al. 2015). Even before that, reforms under the Mental Health Parity and Addiction Equity Act of 2008 (MHPAEA) required commercial health plans, as well as Medicaid managed-care plans, to cover substance abuse treatment services at comparable levels to medical and surgical services. The ACA expands access to health insurance through Medicaid, further promotes insurance parity, and encourages new models of payment and service delivery. Although the MHPAEA and the ACA do not guarantee parity coverage for all Medicaid recipients, they offer a variety of mechanisms by which States may do so at their discretion (Burns 2015). (For more information on the influence of these health care reforms on treatment access, see the sidebar “Parity, the Affordable Care Act, and Access to Treatment.”)

It is notable, however, that empirical studies prior to these reforms did not identify insurance coverage as one of the most significant predictors of entering alcohol treatment (Schmidt and Weisner 2005). Because addiction treatment is heavily subsidized by a separate stream of federal block grant funding, uninsured individuals often appeared to have better access to alcohol treatment than some groups of insured people. The MHPAEA and ACA may be changing this by expanding access to health insurance, deepening mandates for parity, and offering unprecedented opportunities for service growth and delivery-system reform. Under the ACA, overall funding for substance abuse services is increasing (Buck 2011). Before the health care reforms, Medicaid was not a major funder of substance abuse treatment, but this now is changing (Andrews et al. 2015*b*).

The State of Massachusetts, which created the blueprint for the ACA, presents a window into the potential long-range impacts of the federal reforms. This State’s experience paints a cautiously optimistic picture for the Nation. Since the State’s health care reforms, treatment capacity in Massachusetts has expanded to accommodate a growing number of people seeking alcohol services. Treatment admissions increased by 17.1 percent, and daily censuses of patients in substance abuse treatment increased by 4.7 percent. However, the reforms in Massachusetts appear to be having somewhat mixed effects on the quality of care, and uninsured people continue to face challenges (Maclean and Saloner 2015).

In nationwide studies carried out since the passage of the ACA and the MHPAEA, having Medicaid or private insurance was associated with a higher likelihood of receiving substance abuse treatment among people who perceived a need for it (Ali et al. 2015; Mechanic 2012). Moreover, national studies of health plans suggest that the 2008 MHPAEA parity law has met its goal of putting coverage for behavioral health care on par with coverage for medical and surgical care (Horgan et al. 2015). For people with commercial insurance, the MHPAEA has had modest effects on reducing out-of-pocket costs and increasing access to outpatient services (Haffajee et al. 2015). Federal parity also is associated with an increased probability of out-of-network visits and increased average spending on substance abuse treatment (McGinty 2015). Many predicted that, under parity laws, health plans would more aggressively manage utilization, for example, through more stringent requirements on prior authorization for services. However, a national survey of health plans found that only 5 percent of plans require prior authorization for outpatient substance abuse treatment (Merrick et al. 2015).

**Parity, the Affordable Care Act, and Access to Treatment**

Although having insurance coverage is not the most important factor influencing access to substance abuse treatment, the ways in which insurance coverage works do affect treatment availability and influence people’s decisions about seeking care. Recent health care reforms present both fresh opportunities and new barriers affecting treatment access.

The Mental Health Parity and Addiction Equity Act of 2008 requires group health plans offering mental health and addiction services to cover such services at the same levels that they cover other medical and surgical services. The law applies to Medicaid managed-care plans as well as to private plans, but exempts health plans with fewer than 50 employees. Parity technically means that all aspects of coverage are comparable to those covering medical and surgical care, including deductibles and copayments, limitations on the frequency of treatment, and methods of determining whether treatment is necessary. Coverage for alcohol treatment offered by insurance plans therefore becomes more generous under this reform. However, the law does not require that plans cover addiction treatment at all, nor does it require that all areas of addiction be covered. Because of this, there are concerns that companies previously offering some addiction treatment benefits may choose to drop coverage in response to the parity law (Stewart and Horgan 2011).

The Patient Protection and Affordable Care Act of 2010 (ACA) extends insurance coverage to more Americans by expanding Medicaid eligibility and requiring individuals to obtain insurance coverage. Because private insurance plans still are not required to furnish substance abuse coverage, the focus of discussions about access to alcohol and other substance treatment revolves primarily around the effects of the expanded Medicaid benefits. The ACA also includes ideas for health care delivery and payment reforms that are likely to help providers deliver a wider range of behavioral health services. It encourages the use of preventive services, continuity of care, and substance abuse education. It also allows providers treating mental illness to pay more attention to substance abuse problems and provides pathways for incorporating evidence-based treatments. As poor continuity and coordination of care accounted for part of the substance abuse treatment gap and problems with treatment access, the ACA may offer tools to address these issues (Mechanic 2012).

These two pieces of legislation seem to have an impact on the treatment gap. For example, insured people who heretofore ran into caps or limits on their substance abuse coverage may benefit from the parity requirement. In addition, some people who previously could not afford insurance will now be able to obtain coverage (Mark et al. 2011). However, although the ACA does not allow States to reduce Medicaid enrollment, they still can cut health care services funded through general State funds. Because substance abuse treatment relies heavily on non-Medicaid public funds through block grants, treatment and ancillary services remain especially vulnerable to funding cuts during State budget shortfalls (Mark et al. 2011).

ReferencesMarkTLLevitKRVandivort-WarrenRChanges in U.S. spending on mental health and substance abuse treatment, 1986–2005 and implications for policyHealth Affairs3028429220112128935010.1377/hlthaff.2010.0765MechanicDAnalysis and commentary: Seizing opportunities under the Affordable Care Act for transforming the mental and behavioral health systemHealth Affairs3137638220122232316810.1377/hlthaff.2011.0623StewartMTHorganCMHealth services and financing of treatmentAlcohol Research & Health334389394201123580023PMC3860539

Although the evidence to date is promising, a variety of limitations in the implementation of the new laws suggest that it could take many years to realize the promise of federal parity and health care reform. Twenty States have completely opted out of the ACA’s Medicaid expansion program, thus substantially limiting its national impact. There are further concerns that treatment systems may lack the capacity and manpower to treat the swelling numbers of newly covered individuals (Ghitza and Tai 2014; Weil 2015). One survey of State agencies found that fewer than half were helping providers to modernize care or had technical support to maximize insurance participation (Andrews et al. 2015*a*). Similarly, a study of public treatment programs in Los Angeles County found them ill prepared to align their programs with the new realities of health care reform (Guerrero et al. 2015).

## Access to What? New Treatments and Service Delivery Systems

Services research has demonstrated that access to new treatment modalities and service-delivery forms is in flux under health care reform. Service delivery and payment innovations introduced by the ACA could facilitate access to services that have not previously been reimbursable, including comprehensive care management, care coordination, social support, transition care, collaborative care, and other evidence-based interventions. The ACA also has ushered in a trend toward integrating addiction and primary health care under the auspices of “patient-centered medical homes” (PCMH) and Medicaid “health homes” (Starfield and Shi 2004). Health homes target chronic-disease comorbidities prevalent in alcohol treatment populations, and almost all participating States include substance abuse in their qualifying conditions.

The PCMH model originated in private health plans as a strategy to lower costs while improving the quality and continuity of care. Under this model, substance abuse services are linked to primary care through strong referral networks using electronic medical records, or they may be “co-located” under one roof in efforts to more deeply integrate care (Rittenhouse and Shortell 2009). Early evaluations—mostly in large, integrated delivery systems—show that this model improves quality, with savings in total health care costs (Crabtree et al. 2011). To a more limited extent, PCMH applications have shown positive outcomes for accessibility and continuity of care in safety-net populations, where substance abuse treatment need is disproportionately high (Rittenhouse et al. 2012).

Health care reform further appears to be catalyzing a longstanding structural shift toward the use of screening and brief interventions (SBIs) delivered in mainstream medical care settings, most notably primary care and hospital settings (Babor and Higgins-Biddle 2000). SBIs may help close the treatment gap by expanding capacities within mainstream medical care settings. An SBI can be as brief as 5 to 10 minutes and can be particularly effective when performed by a primary care physician. It begins with an assessment of the patient’s alcohol use; patients screening positive for an alcohol problem then are advised to cut down or abstain and may be referred for further professional help. Studies have long shown that SBI offers an evidence-based, cost-effective approach for reducing patients’ drinking (Fleming and Barry 1991). Introducing SBI programs into settings such as Federally Qualified Health Centers,^2^ schools, workplaces, and criminal justice settings could broaden their reach and also help more disadvantaged populations (Mulia et al. 2014). Health services researchers are developing and testing more streamlined Web-based approaches to training health care providers in SBI skills, which could increase the system’s capacity to provide this form of care (Stoner et al. 2014). Electronic versions of SBI and “guided self-change” approaches also hold promise for allowing efficient self-treatment for people with moderately severe substance use disorders (Sinadinovic et al. 2014; Wagner et al. 2014). However, a 2010 national survey of health plans found that only 18 percent of insurance products required screening for alcohol- and drug-abuse problems in primary care (Garnick et al. 2014).

A related challenge is promoting the adoption of even newer evidence-based treatments, most notably pharmaceutical approaches. “Second-generation” medications, such as acamprosate and regular and extended-release naltrexone, are clinically efficacious during detoxification and recovery from alcohol abuse. A national survey of health plans found that 96 percent of insurance products included coverage for addiction medications (Horgan et al. 2014). However, for patients, difficulties in gaining health plan authorization and covering high copayments may be barriers to using addiction medications. Providers also face challenges ordering and obtaining licenses to administer certain medications.

Initiatives such as Advancing Recovery and the Medication Research Partnership have been effective in working with the public and private sectors to facilitate adoption of pharmacotherapies for AUD. These organizational-change initiatives bring payers and providers together into collaboratives that test organizational changes supporting the increased use of medications through brief, experimental “change cycles.” Implementation strategies that work are quickly scaled up through sharing across members of the collaborative. Demonstrations suggest that supported partnerships such as these can achieve a wider adoption of evidence-based treatment practices more rapidly and effectively (Ford et al. 2015; Schmidt et al. 2012).

## Bridging the Treatment Gap: A Continuing Agenda

As seen through the lens of health services research, problem drinkers face better prospects for treatment in the current landscape, characterized by the expansion of insurance coverage under health care reform and parity laws, as well as rapid clinical innovations and service-delivery-system reforms. But it also is a landscape in which the need for care still far outstrips the supply of treatment—one in which waiting lists for care are long as the alcohol field looks to the wider health care system to build greater capacity. Above all, today’s health services researchers describe a treatment system that is moving toward closer alignment with the wider health care system. This can be seen in the movement toward more integrated models of service delivery through the PCMH and Medicaid health homes. It also is evident in the push toward parity in insurance coverage, and in the scaling-up of SBI programs in primary care and other medical care settings. Finally, alignment with the greater health care system can be observed in the promotion of pharmaceutical therapies, most notably the new second-generation pharmaceuticals for treating addiction. Deepening collaboration between alcohol treatment and mainstream health care systems will likely lead to further—undoubtedly controversial—changes in services for people with alcohol problems. But this may very well be the field’s best hope for solving what is arguably its greatest challenge: reaching a greater proportion of the population in need of care.

## Footnotes

1 According to the 2014 NSDUH, the prevalence of abuse or dependence among men was 3.4 percent for illicit substances, 8.5 percent for alcohol, and 10.7 percent for illicit drugs or alcohol, compared with 1.9 percent, 4.4. percent, and 5.7 percent, respectively, among women (Center for Behavioral Health Statistics and Quality 2015).

2 Federally Qualified Health Centers are community-based organizations that offer comprehensive primary care and preventive care, including substance abuse services, to people of all ages, regardless of their ability to pay or health insurance status. They are therefore an important part of the health care safety net.
